# Endothelial MICU1 alleviates diabetic cardiomyopathy by attenuating nitrative stress-mediated cardiac microvascular injury

**DOI:** 10.1186/s12933-023-01941-1

**Published:** 2023-08-17

**Authors:** Xide Shi, Chao Liu, Jiangwei Chen, Shiqiang Zhou, Yajuan Li, Xingcheng Zhao, Jinliang Xing, Junhui Xue, Fengzhou Liu, Fei Li

**Affiliations:** 1 Department of Cardiology, Xijing Hospital, The Fourth Military Medical University, Xi’an, China; 2 State Key Laboratory of Oral & Maxillofacial Reconstruction and Regeneration, National Clinical Research Center for Oral Diseases, Shaanxi Clinical Research Center for Oral Diseases, Department of Medical Rehabilitation, School of Stomatology, The Fourth Military Medical University, Xi’an, China; 3 Aerospace Clinical Medical Center, School of Aerospace Medicine, The Fourth Military Medical University, 169 Changle West Road, Xi’an, 710032 Shaanxi China; 4 Department of Physiology and Pathophysiology, State Key Laboratory of Cancer Biology, The Fourth Military Medical University, Xi’an, China; 5 Department of Aviation Medicine, Xijing Hospital, The Fourth Military Medical University, Xi’an, China

**Keywords:** MICU1, Diabetic cardiomyopathy, Cardiac microvascular endothelial cells (CMECs), Nitrative stress, Inflammatory response, Endothelial permeability

## Abstract

**Background:**

Myocardial microvascular injury is the key event in early diabetic heart disease. The injury of myocardial microvascular endothelial cells (CMECs) is the main cause and trigger of myocardial microvascular disease. Mitochondrial calcium homeostasis plays an important role in maintaining the normal function, survival and death of endothelial cells. Considering that mitochondrial calcium uptake 1 (MICU1) is a key molecule in mitochondrial calcium regulation, this study aimed to investigate the role of MICU1 in CMECs and explore its underlying mechanisms.

**Methods:**

To examine the role of endothelial MICU1 in diabetic cardiomyopathy (DCM), we used endothelial-specific MICU1^ecKO^ mice to establish a diabetic mouse model and evaluate the cardiac function. In addition, MICU1 overexpression was conducted by injecting adeno-associated virus 9 carrying MICU1 (AAV9-MICU1). Transcriptome sequencing technology was used to explore underlying molecular mechanisms.

**Results:**

Here, we found that MICU1 expression is decreased in CMECs of diabetic mice. Moreover, we demonstrated that endothelial cell MICU1 knockout exacerbated the levels of cardiac hypertrophy and interstitial myocardial fibrosis and led to a further reduction in left ventricular function in diabetic mice. Notably, we found that AAV9-MICU1 specifically upregulated the expression of MICU1 in CMECs of diabetic mice, which inhibited nitrification stress, inflammatory reaction, and apoptosis of the CMECs, ameliorated myocardial hypertrophy and fibrosis, and promoted cardiac function. Further mechanistic analysis suggested that MICU1 deficiency result in excessive mitochondrial calcium uptake and homeostasis imbalance which caused nitrification stress-induced endothelial damage and inflammation that disrupted myocardial microvascular endothelial barrier function and ultimately promoted DCM progression.

**Conclusions:**

Our findings demonstrate that MICU1 expression was downregulated in the CMECs of diabetic mice. Overexpression of endothelial MICU1 reduced nitrification stress induced apoptosis and inflammation by inhibiting mitochondrial calcium uptake, which improved myocardial microvascular function and inhibited DCM progression. Our findings suggest that endothelial MICU1 is a molecular intervention target for the potential treatment of DCM.

**Supplementary Information:**

The online version contains supplementary material available at 10.1186/s12933-023-01941-1.

## Background

Diabetes is a major disease that seriously endangers human health. It is estimated that the global prevalence of diabetes will reach 10.2% (578 million) by 2030 [1]. Cardiovascular complications are the leading cause of morbidity and mortality in patients with diabetes and account for an estimated 80% of deaths in these patients [2]. Growing evidence indicates that cardiac microvascular endothelial dysfunction is a key event in diabetic myocardial microvascular disease throughout the entire course of diabetic cardiomyopathy (DCM) [3–5]. For example, the exposure of cardiac microvascular endothelial cells (CMECs) to hyperglycemia for a long time induced energy metabolism disorders, oxidative/nitrifying stress, and inflammatory reactions and led to myocardial microvascular damage in diabetes [4, 6, 7]. Although increasing evidence suggests a link between endothelial mitochondrial dysfunction and myocardial microvascular injury in patients with diabetes, the underlying mechanisms of these processes are not clear.

MICU1 is a key molecule that regulates mitochondrial calcium homeostasis, which is closely related to myocardial injury in diabetes [8–10]. Our previous studies showed that the upregulation of MICU1 expression by injecting MICU1-expressing adenovirus (Ad-MICU1) into the myocardium protected the heart against DCM in *db/db* mice [11]. This study substantiated the role of MICU1 in DCM, but it raised a number of questions.

Because the Ad-MICU1 injected into the myocardium of *db/db* mice was non-specific in our previous work, the expression of MICU1 in multiple cardiac cells may also be up-regulated after injection. Therefore, we could not determine whether the improvement in cardiac function was caused by the reconstruction of MICU1 expression in cardiomyocytes or the combined effect of the upregulation of MICU1 expression in multiple cardiac cells. MICU1 serves as a molecular gatekeeper to prevent mitochondrial calcium overload, reduce oxidative stress, and maintain normal cell function [12, 13]. However, whether MICU1 participates in the development of myocardial microvascular injury in diabetes by affecting the function of CMECs has not been reported.

Our preliminary experimental results demonstrated that the expression level of MICU1 in CMECs of diabetic *db/db* mice was significantly reduced and the upregulation of MICU1 expression inhibited the apoptosis of CMECs isolated from diabetic mice. Does the down-regulation of MICU1 in CMECs participate in the process of DCM? Can the upregulation of MICU1 improve myocardial microvascular injury in diabetes? These questions remain unanswered, but further attempts to investigate the potential underlying mechanisms will undoubtedly provide a breakthrough in the prevention and treatment of DCM. Therefore, the present study examined the role of the decreased expression of MICU1 in CMECs in DCM and its related mechanisms.

## Materials and methods

### Animals

All male C57BL/6 mice were obtained from the animal center at the Fourth Military Medical University. Receptor-deficient C57BLKS (*db/db*) mice and wild-type C57BLKS mice were purchased from Beijing Vital River Animal Laboratory (Beijing, China). MICU1^flox/flox^ (C57BL/6 background) mice were generated by Guangzhou Cyagen Company (Guangzhou, China). Mice with an endothelial-specific knockout of MICU1 (MICU1^ecKO^ mice) were generated by crossing Cdh5-Cre transgenic mice with MICU1^flox/flox^ mice. All animal experiments followed the Guidelines for Animal Protection and Use of the People’s Republic of China and were approved by the Animal Ethics Committee of the Air Force Military Medical University. All experimental mice were raised in the Animal Experiment Center of Air Force Military Medical University under standard pathogen-free conditions at 20–23 °C and a 12-h light-dark cycle. The experimental mice were randomly divided and blinded in all in vivo experiments and subsequent assessments. MICU^flox/flox^ mice and MICU1^ecKO^ mice aged 4–5 weeks were used in the in vitro cell experiments, and the primary CMECs were divided for subsequent experiments.

### Identification of mouse tail genotype

A One Step Mouse Genotyping Kit (Vazyme Biotech, Nanjing, China, PD101-01) was used to extract the DNA of the target mouse according to the instructions of the reagent manufacturer. The DNA concentration was determined, and PCR amplification of the mouse tail gene was performed according to the instructions of the kit (See Table 1 of Supplementary Materials for PCR primers). The size of the DNA fragments was identified using agarose gel electrophoresis, and an ultraviolet photography system was used to capture photos and analyze and determine the mouse genotype.

**Table 1:** PCR primers

Gene Name	Forward Primer	Reverse Primer
MICU1^loxp/loxp^	GAACTAGCCAAGATAAACAGCGTG	GTGTGTTTCCTGTGGTGGTCTG
CDH5-cre	CCAGGCTGACCAAGCTGAG	CCTGGCGATCCCTGAACA
iNOS	TGGAGCCAGTTGTGGATTGTC	GGTCGTAATGTCCAGGAAGTAG
IL-1β	CAGCACATCAACAAGAGCTTCAG	GAGGATGGGCTCTTCTTCAAAGA
TNF-α	GACCCTCACACTCAGATCATCTT	CCTTGAAGAGAACCTGGGAGTAG
ICAM-1	AAGGGCTGGCATTGTTCTCTAAT	GCCTCCCAGCTCCAGGTATAT
NF-κB	CTTCTGGGCCTTATGTGGAGATC	GGTCCTGTGTAGCCATTGATCTT
NLRP3	AGATGGAGAAGGCAGATCACTTG	CAAATGGAGATGCGGGAGAGATA
MICU1	ACAACAGTCCTCTCCACTCCTCAG	CCTCCATGTCTACCTCTCCGTCTC
β-actin	AGCCATGTACGTAGCCATCCA	TCTCCGGAGTCCATCACAATG

### Experimental mouse diabetes model

Male mice aged 4 weeks were fed a high-fat diet (HFD, 45% kcal fat) for 4 weeks, and streptozotocin (STZ; 100 mg/kg; Sigma, USA; S0130) was intraperitoneally injected. STZ was dissolved in 4 °C precooled citric acid buffers prepared in advance, pH 4.2–4.5. STZ was dissolved in the dark, and the injection was completed within 30 min after dissolution. Blood glucose ≥ 15.0 mmol/L 48 h after the injection indicated successful construction of the diabetes model. The control group mice were intraperitoneally injected with the same amount of citric acid buffer (carrier) after fasting. At this point, the mice were approximately 8–9 weeks old and continued to be fed a high-fat diet for 6 weeks. AAV9-GFP and AAV9-MICU1 were injected into the myocardium of 14-week-old diabetic mice. Subsequently, echocardiography was used to evaluate the cardiac function of diabetic MICU1^ecKO^ mice (18 weeks old) after intramyocardial injection of AAV9-MICU1 or AAV9-GFP. Haematoxylin and eosin staining and wheat germ agglutinin staining were used to evaluate the pathological changes of diabetic MICU1^ecKO^ mice (18 weeks old). Intraperitoneal Glucose tolerance tests (IPGTTs; 2 g/kg glucose) and insulin tolerance test (ITTs; 0.75 units/kg insulin) were monitored at the age of 17–18 weeks according to our previously reported method.

### Isolation, culture and identification of CMECs

CMECs were isolated as previously described with slight modifications [14]. Male mice aged 4–5 weeks (WT or MICU1^ecKO^ mice) were euthanized via isoflurane inhalation, and the cardiac apex and left ventricle were dissected, minced, and incubated in 4 ml of 0.2% type II collagenase (Gibco, USA; 17,101,015) for 10 min at 37 °C. The same volume of 0.25% trypsin (Solarbio, T1350) was added and digested for 5 min. The digestion was terminated by the addition of DMEM containing 20% FBS (Gibco, USA; 10,100,147). After several minutes of incubation, the supernatant was absorbed and transferred to a sterile centrifuge tube for centrifugation and discarded. The CMECs were resuspended in complete medium (DMEM, with 20% FBS) and cultured continuously in a thermostatic incubator (5% CO_2_, 37 °C). The culture medium was changed at 6 and 24 h then every 2–3 days. After approximately 8–10 days, when the cells fused to approximately 80% confluence, the cells were digested and subcultured. CMECs were identified using the specific CD31 monoclonal antibody immunofluorescence method. Cells labeled by CD31 were identified as CMECs. The purity of CMECs was greater than 90%. Third-generation cells were selected for the experiment. The concentration of glucose in normal culture was 5.5 mmol/L. For high glucose and high fat (HGHF) treatment, CMECs were treated with DMEM containing 33.3 mmol/L glucose and 500 mmol/L sodium palmitate (Sigma, USA; P9767) for 24 h (according to our previously established method) [11].

### Forced expression of MICU1

Mouse MICU1-overexpressing adeno-associated virus-9 (AAV9-MICU1) genome particles containing the endothelial-specific TIE promoter and Flag and the control virus carrying GFP (AAV9-GFP) were constructed by Hanbio Technology (Shanghai, China). Mice were injected with 5 × 10^11^ viral particles (vp) of AAV9-GFP or AAV9-MICU1 via myocardial injection, as previously described [11]. The transfection efficiency was evaluated using immunofluorescence and Western blotting 4 weeks after AAV9 injection. CMECs were infected with MICU1 adenovirus (Ad-MICU1) or control adenovirus (Ad-EV), which was constructed by Hanbio Technology (Shanghai, China). The specific infection method was described previously [11]. The cells were cultured in normal glucose medium (NG, 5.5 mmol/l) or HGHF medium for another 24 h then used for further analysis.

### Echocardiography

As described previously [15], the Vevo 2100 ultrasound system (VisualSonics, Toronto, Ontario, Canada) was used to evaluate the cardiac function of mice. Briefly, the mice were lightly anesthetized with isoflurane (3% for induction and 1% for maintenance), and the cardiac function of the mice was detected using a hand-held ultrasonic operation transducer. After obtaining echocardiography images, the left ventricular fractional shortening (LVFS), left ventricular diastolic diameters (LVIDd) and left ventricular posterior wall thickness in diastole (LVPWd) of the mice hearts were analyzed using Vevo 2100 software. The same researcher who was blinded to the treatment analyzed all variables and data.

### Immunofluorescence staining

Frozen sections were fixed with 4% paraformaldehyde for 20 minutes and permeabilized with 0.2% Triton (Sigma, X-100) at room temperature (RT) for 20 minutes. Before FITC-Lectin staining + CD31 staining, we used a fluorescence quenching agent (G1221, Servicebio, China) to quench the fluorescence of GFP transfected with the AAV in myocardial tissue. After washing with PBS, the slices were blocked with 2% bovine serum albumin (BSA) at RT for 1 hour. The samples were incubated with the primary antibodies overnight at 4°C and the appropriate fluorescent secondary antibodies at RT for 1 hour (away from light). The nuclei were stained with 4’,6-diamidino-2-phenylindole (DAPI). Images were captured under a confocal microscope (Olympus FV3000, Japan). Captured images were quantitatively analyzed using ImageJ (version 1.53c, NIH, USA). The primary antibodies for immunoblotting are listed in Table 2 of the Supplementary Materials.

**Table 2:** Primary antibodies used in western blot and immunofluorescent staining

Name	Manufacturer	Cat No.	Dilution	Host	Application
MICU1	CST	12524s	1:1000	Rabbit	WB
MICU2	Abcam	Ab101465	1:1000	Rabbit	WB
MCU	Abcam	ab219827	1:500	Mouse	WB
MCUR1	Boster	A08547-1	1:1000	Rabbit	WB
p-eNOS	Abclonal	AP0421	1:1000	Rabbit	WB
iNOS	Abclonal	A0312	1:1000	Rabbit	WB
Caspase 1	Immunoway	YT5743	1:1000	Rabbit	WB
NF-κB	Abclonal	AP0475	1:1000	Rabbit	WB
NLRP3	Abclonal	A5652	1:1000	Rabbit	WB
IL-1β	Abclonal	A16288	1:1000	Rabbit	WB
eNOS	Abclonal	A15075	1:1000	Rabbit	WB
Cleaved caspase 1	CST	89,332 S	1:1000	Rabbit	WB
TNF-α	Abclonal	A11534	1:1000	Rabbit	WB
β-actin	Abclonal	AC026	1:5000	Rabbit	WB
CD31	Abcam	ab7388	1:400	Rat	IHC-Fr, ICC/IF
VE-cadherin	Abcam	Ab205336	1:500	Rabbit	ICC/IF
ICAM-1	Abclonal	A5597	1:100	Rabbit	ICC/IF
Donkey anti-Rat AF488	Thermo Fisher	A21208	1:500	Donkey	ICC/IF
Donkey anti-Rat AF594	Thermo Fisher	A21209	1:500	Donkey	IHC-Fr, ICC/IF
Donkey anti-Rabbit AF594	Thermo Fisher	A21207	1:500	Donkey	IHC-Fr, ICC/IF

### Measurement of microvascular perfusion

As previously described [16], FITC-conjugated tomato lectin (Sigma, USA) was injected into mice via the tail vein and allowed to circulate for 10 min. Hearts were collected, sectioned and stained with CD31 to label blood vessels. The quantification of microvascular perfusion was expressed as the percentage of FITC-lectin-positive microvessels and CD31-positive microvessels.

### Histological analysis

Mouse hearts were fixed in 4% paraformaldehyde (PFA) for 48 h and paraffin embedded. Standard hematoxylin and eosin staining was performed according to standard procedures [17]. Masson trichrome staining was performed following the instructions of the Masson staining kit (Solarbio, China, G1346). The cross-sectional area of myocardial cells was detected using wheat germ agglutinin staining according to the instructions of the kit (WGA, Sigma, USA, red).

### Transcriptome sequencing technology

Transcriptome sequencing technology was supported by Tsingke Biotechnology (Beijing, China). The isolated primary CMECs of the WT and MICU1^ecKO^ mice were used for the RNA-seq experiment after 24 h of culture in normal or HGHF conditions. The qualified total RNA was extracted, followed by mRNA purification and reverse transcription after fragmentation, and the cDNA library was constructed. The screening criteria were log^2^ |fold change|≥ 2.0 and *p* value < 0.05.

### Western blotting

According to the manufacturer’s instructions, cells were routinely harvested and lysed in RIPA buffer (Beyotime, China) to extract protein. The protein concentration was measured using the BCA method and equalized before loading. Approximately 30 µg of total protein samples were added to SDS‒PAGE gels and transferred to PVDF membranes (Millipore, USA). 5% skimmed milk powder was incubated at RT for 1 h, and a properly diluted primary antibody was added and incubated overnight at 4 °C. After 30 min of rewarming, membranes were washed with TBST three times for 7 min each time. After incubation with the secondary antibody at RT for 1 h, TBST was used to wash the membrane, and a chemiluminescence system (Bio-Rad) was used to detect the expression of each protein. The results were quantified using Image J software (version 1.53c, NIH, USA). The antibodies are shown in Table 1 of the Supplementary Materials.

### Quantitative real-time PCR

Total RNA from cells was extracted using a Tiangen Total RNA Extraction Kit (Tiangen, China, DP424). The concentration and purity of total RNA were detected after extraction. Reverse transcription of 500 ng RNA into cDNA was performed according to the instructions of the TAKARA Reverse Transcription Kit (TaKaRa, Japan, RR047A). SYBR was detected according to the instructions of the ® Premix Ex Taq™ II reagent kit (TaKaRa, Japan, RR820A) in a CFX96 Real Time PCR Detection System (Bio Rad) instrument. The following reaction conditions were used: 95℃ for 30 s; 40 cycles at 95℃ for 10 s, 56℃ for 30 s and 72℃ for 30 s; 72℃ for 5 min. Each sample was inserted into 3 holes, and the experiment was repeated 3 times. The 2^-ΔΔCt^ method was used to analyze and process the data (See Table 2 of Supplementary Materials for specific primer sequences).

### Mitochondrial calcium concentration determination

According to the manufacturer’s instructions, the fluorescent compound dye Rhod-2-AM (Molecular Probes) was used to monitor the mitochondrial Ca^2+^ concentration in CMECs treated under different conditions. CMECs were viewed with a confocal laser scanning microscope (Olympus FV3000, Japan) as described previously [11].

### Detection of CMECs apoptosis

Heart tissue samples were treated as described previously [18]. CD31 and TUNEL assay (Sigma, USA) double immunofluorescence staining was performed to measure CMECs apoptosis in heart tissue. Fluorescent staining was visualized using a confocal laser scanning microscope (Olympus FV3000, Japan). For cell samples, a biological apoptosis test kit (BestBio, China, BB-4101) was used for detection. The pancreatic enzyme without EDTA was used to digest the CMECs, and the treated cells were collected with the medium into a 15 ml centrifuge tube. The tubes were centrifuged at 1000 rpm for 5 min and washed twice in precooled PBS. Binding Buffer binding solution (400 µl) was added and mixed, and 10 µl Annexin V-FITC was added for incubation in the dark at RT for 15 min. 7 µl PI dye was added, gently mixed, and incubated in the dark at 4℃ for 5 min. Flow cytometry was used for detection and analysis.

### Measurement of CMECs permeability in vitro

To measure CMECs permeability, cells were grown to confluence on ThinCertTM cell culture inserts with a pore diameter of 0.4 μm to form a fused monolayer in a 24-well plate (Corning, USA). As previously described [19], cells were incubated with FITC-dextran (1 mg/mL) in the medium at a constant temperature of 37 °C. Four hours later, the relative fluorescence passing through the chamber was determined to evaluate the degree of permeability using a ceramic galaxy microplate reader (BMG Labtechnologies, Germany, excitation 485 nm, emission 520 nm).

### Mitochondrial calcium clearance

As previously described [11], we constructed an adenovirus that carried parvalbumin encoding mitochondrial Ca^2+^-binding protein (Ad-mitoPV) for overexpression in CMECs. After the infection of CMECs with Ad-mitoPV (30 MOI), the overexpression of parvalbumin in the mitochondria of CMECs bound Ca^2+^ and reduced the calcium content of mitochondria.

### Determination of 3-nitrotyrosine

3-Nitrotyrosine (3-NT) was determined using the 3-Nitrotyrosine ELISA Kit (Abcam, USA, ab116691) according to the manufacturers’ instructions. CMECs were routinely digested, collected and centrifuged at 4 °C for 10 min at 500 g. The cells were rinsed twice with PBS. The cell particles in the extract were solubilized at a rate of 2 × 10^7^/mL. The cells were incubated on ice for 20 min and centrifuged at 16,000 × g at 4 °C for 20 min. The supernatant was transferred to a clean tube for detection. The specific operation method was performed according to the instructions of the ELISA kit.

### Nitration product removal

We used FeTMPyP (Cayman, Ann Arbor, USA) to clear the nitration products. FeTMPyP is an ONOO- degradation catalyst that significantly reduces the formation of mitochondrial nitrite [11]. The experimental group was incubated with FeTMPyP (50 µM) in the cell culture medium for 24 h for subsequent detection.

### Mitochondrial membrane potential detection

According to the manufacturer’s protocol, mitochondrial membrane potential changes were evaluated using a JC-1 mitochondrial membrane potential detection kit (Beyotime, China). Briefly, CMECs were stained with JC-1 reagent at 37 °C for 30 min and washed three times with PBS. The changes in mitochondrial membrane potential were measured via flow cytometry. The red fluorescence intensity represented the strength of the mitochondrial membrane potential.

### Statistical analysis

SPSS 23.0 statistical software was used for analysis. Continuous measurement data with a normal distribution are expressed as the means ± SEM. One-way analysis of variance (ANOVA) was used for comparisons between multiple groups, and the Bonferroni post hoc test was used for correction. Student’s *t* test was used for comparisons between two groups, and the LSD-*t* test was used for comparisons between multiple groups. The normality test was used for data with n ≥ 6, and Pearson correlation analysis was used for correlations between measurement variables. Fisher’s exact test was used to compare classification variables, and GraphPad Prism 8.3.0 (GraphPad Software, Inc., USA) was used for statistical analyses. *P* < 0.05 was considered a statistically significant difference.

## Results

### MICU1 expression is decreased in CMECs of diabetic mice

**Fig. 1:**
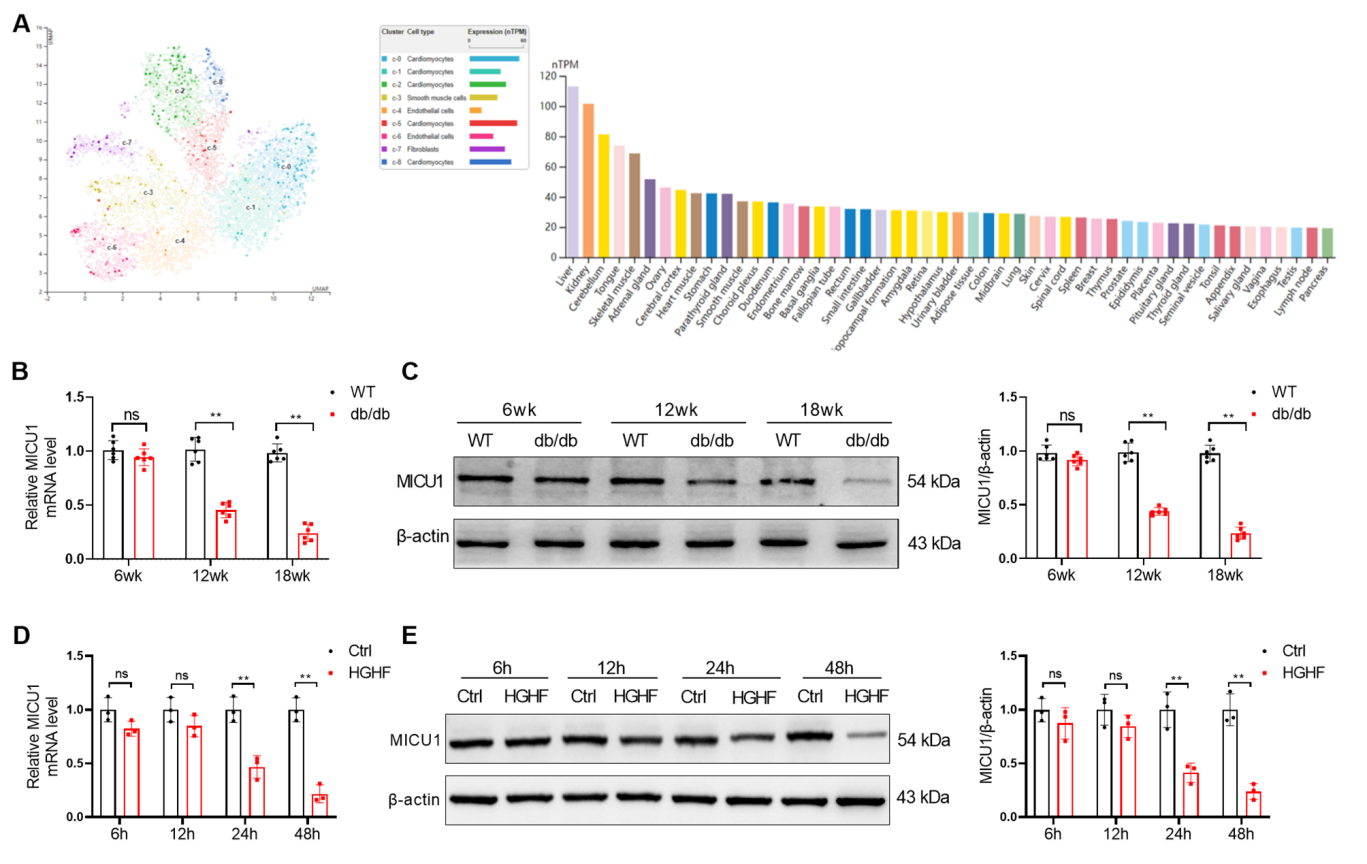
MICU1 expression is decreased in CMECs of diabetic mice. (**A**) Cell type-specific MICU1 mRNA expression in different organ tissues and hearts of adults. The MICU1 mRNA levels in different organ tissues and hearts of adults were determined by mining single-cell RNAseq data deposited in the Human Protein Atlas database (https://www.proteinatlas.org/ENSG00000107745-MICU1/single+cell+type/heart+muscle). Different cardiac cell types are colored according to clusters and expressed by uniform manifold approximation and projection. (**B**) qRT-PCR analysis of MICU1 mRNA levels in the CMECs of diabetic *db/db* mice and wild-type littermate mice (n = 6, 6-, 12- and 18-week-old mice per group). (**C**) Western blotting analysis of the protein expression levels of MICU1 and β-actin in the CMECs of diabetic *db/db* mice and wild-type littermate mice (n = 6, 6-, 12- and 18-week-old per group). (**D**) qRT-PCR analysis of MICU1 mRNA levels in the CMECs treated with HGHF (n = 3 per group). (**E**) Western blotting analysis of MICU1 expression in CMECs treated with HGHF (n = 3 per group), **p < 0.01

MICU1 is involved in the occurrence and progression of various diseases via multiple mechanisms [9, 10]. Previous studies found that MICU1 was downregulated in the cardiomyocytes of diabetic mice and involved in diabetic myocardial injury by regulating the redox status of cardiomyocytes [11]. In a complex disease, such as diabetes, it is far from certain that these cardiovascular pathologies all solely result from hyperglycemia [1, 2]. Relevant studies found that MICU1 is essential for normal function of endothelial cells by maintaining mitochondrial calcium homeostasis [12, 13, 22]. To examine whether MICU1 was dysregulated in CMECs and contributed to DCM, we initially analyzed cell-type MICU1 expression in different organ tissues and hearts of adults in The Human Protein Atlas (https://www.proteinatlas.org/ENSG00000107745-MICU1/single+cell+type/heart+muscle). We found that the expression of MICU1 in cardiac endothelial cells was in a high expression state (Fig. 1A). Therefore, we further investigated the expression of MICU1 in CMECs and the effect of HGHF on the expression of MICU1 in CMECs of diabetic *db/db* mice. To confirm the changes of MICU1 expression in vascular endothelial cells of diabetic mice, we isolated the CMECs of 6-, 12- and 18-week-old diabetic *db/db* mice and measured the expression of MICU1 using qRT-PCR and Western blotting. mRNA expression patterns were observed using qRT‐PCR (Fig. 1B), which indicated that the expression of MICU1 in CMECs of diabetic *db/db* mice was primarily regulated at the transcriptional level. Western blotting (Fig. 1C) also demonstrated that MICU1 was significantly downregulated in CMECs of diabetic *db/db* mice at 12 and 18 weeks of age compared to the corresponding controls. The downregulation of MICU1 was more obvious in the CMECs of 18-week-old diabetic *db/db* mice. The expression of MICU1 in CMECs incubated with HGHF was significantly reduced on qRT-PCR (Fig. 1D) and Western blots (Fig. 1E). These data show that MICU1 downregulation occurs in the CMECs of diabetic mice.

### Endothelial-specific knockout of MICU1 exacerbates diabetes-induced cardiac dysfunction in mice

**Fig. 2:**
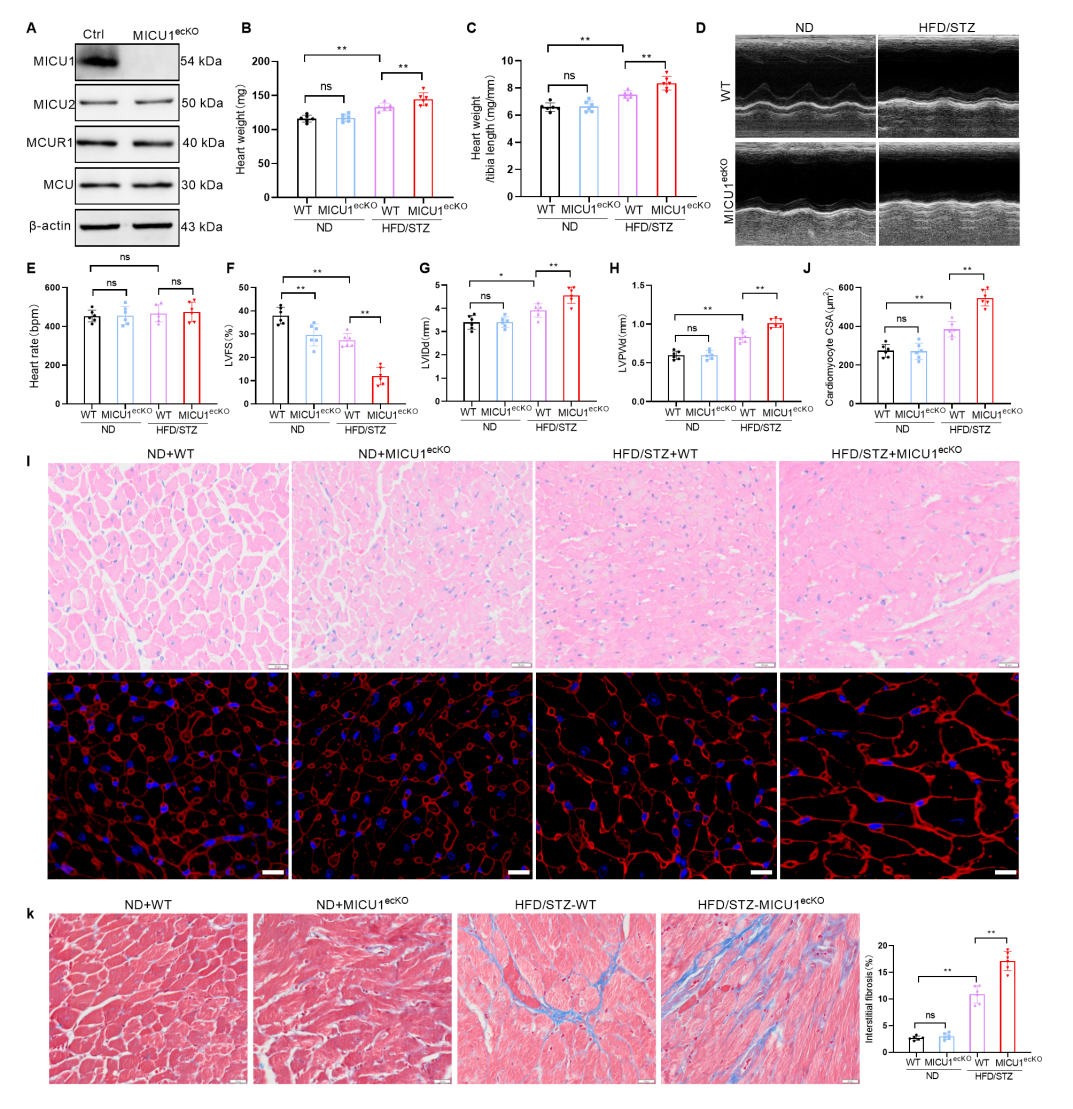
Endothelial-specific knockout of MICU1 aggravates diabetes-induced cardiac dysfunction in mice. (**A**) Western blotting for protein levels of MICU1 in CMECs of WT and MICU1^ecKO^ mice (aged 18 weeks). (**B**) The heart weight of different groups. (**C**) The ratios of heart weight to tibia length (HW/TL) for different groups. (**D**) Representative echocardiographic images of the LV in different 18-week-old mice. (**E**, **F**, **G** and **H**) Echocardiographic assessment of heart rate (E), Echocardiographic assessment of LV fractional shortening (LVFS; F), LV end diastolic diameter (LVIDd; G) and LV diastolic posterior wall thickness (LVPWd; H) in different mice (n = 6, 18 weeks old per group). (**I**) Cardiomyocyte cross-sectional area was analyzed using hematoxylin and eosin staining and wheat germ agglutinin staining in hearts from different mice (n = 6, 18 weeks old per group); scale bar = 20 μm. (**J**) Comparative quantitative analysis of cardiomyocyte CSA. (**K**) Representative images and comparative quantitative analysis of Masson trichrome staining of hearts from different mice (n = 6, 18 weeks old per group); scale bar = 20 μm. *p < 0.05, **p < 0.01

To further examine the functional role of the downregulation of MICU1 expression in CMECs in DCM, we generated endothelial cell-specific MICU1-deficient mice (MICU1^ecKO^ mice) using Cdh5-Cre (Fig. S1). We successfully established a mouse model of type 2 diabetes using a high-fat diet (HFD) in combination with STZ. Then we continue to feed them with HFD until 18 weeks of age. To assess the progression of diabetes in HFD/STZ-MICU1^ecKO^ mice at the ages of 18 weeks, the intraperitoneal glucose tolerance tests (IPGTTs) and insulin tolerance tests (ITTs) were performed as shown in Fig. S2, and the results showed that HFD/STZ-MICU1^ecKO^ mice had higher blood glucose level and lower clearance efficiency of plasma glucose than HFD/STZ-WT mice (Fig. S2). To confirm endothelial cell MICU1 knockout, we isolated primary CMECs and detected the expression of MICU1. As shown in Fig. 2A, the expression of MICU1 was almost undetectable in the endothelial conditional MICU1 knockout mice (MICU1^ecKO^ mice), and mitochondrial calcium regulatory molecules, such as MICU2, MCUR1 and MCU, were expressed normally, and there was no significant change compared with the WT mice (Fig. 2A). Heart weight and the ratio of heart weight to tibia length (HW/TL) were not changed in MICU1^ecKO^ mice compared to WT mice. Knockout of MICU1 further increased heart weight and HW/TL in diabetic MICU1^ecKO^ mice (Fig. 2B, C). Representative M-mode echocardiograms are shown in Fig. 2D. LV fractional shortening were impaired in MICU1^ecKO^ mice compared to WT mice, while there was no significant change in heart rate among all groups (Fig. 2E). Knockout of MICU1 further reduced the LV fractional shortening in diabetic MICU1^ecKO^ mice (Fig. 2F). However, diabetic MICU1^ecKO^ mice showed increased LV end diastolic diameter (LVIDd) and LV diastolic posterior wall thickness (LVPWd) (Fig. 2G, H,). Hematoxylin and eosin staining and wheat germ agglutinin staining showed that the cross-sectional area of cardiomyocytes in diabetic MICU1^ecKO^ mice was significantly increased compared to diabetic mice (Fig. 2I, J). Compared to the WT mice, the diabetic mice showed increased myocardial fibrosis, as indicated by Masson trichrome staining. Knockout of MICU1 further exacerbated the interstitial myocardial fibrosis level of diabetic MICU1^ecKO^ mice (Fig. 2K). Taken together, these data suggested that endothelial cell MICU1 knockout aggravated the levels of cardiac hypertrophy and interstitial myocardial fibrosis and led to a further reduction in left ventricular function in diabetic mice.

### Endothelial-specific knockout of MICU1 exacerbates cardiac microvascular injury in diabetic mice

**Fig. 3:**
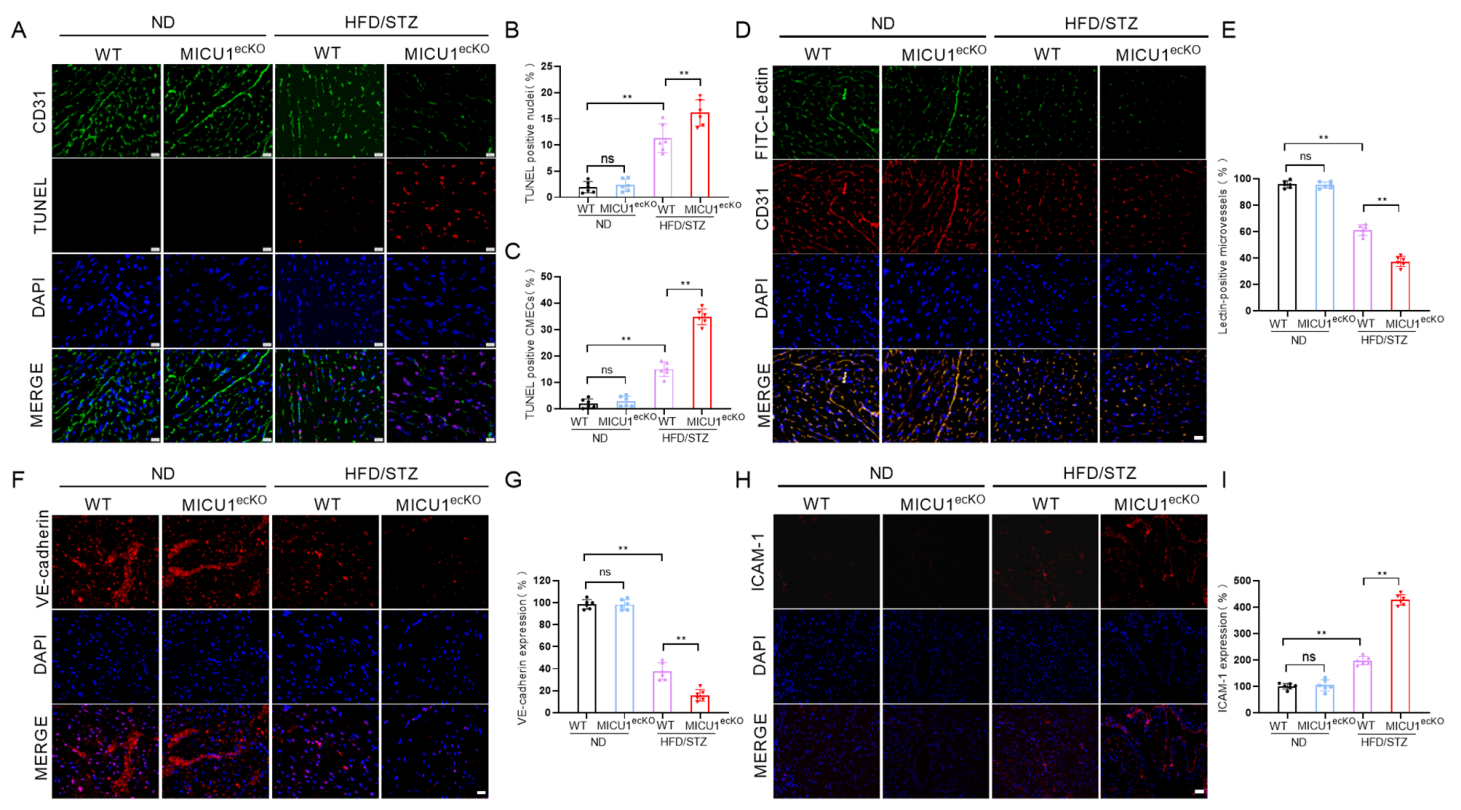
Endothelial-specific knockout of MICU1 exacerbates cardiac microvascular injury in diabetic mice. (**A**) A TUNEL assay was performed to assess CMECs apoptosis, scale bar = 20 μm. (**B**) Percentages of TUNEL-positive nuclei over total number of nuclei (n = 6 per group). (**C**) Percentages of TUNEL positive CMECs over total number of CD31-positive nuclei (n = 6 per group). (**D**) Cardiac microvascular perfusion is indicated by the ratio of lectin-positive microvessels (green) to CD31-positive microvessels (red) (n = 6 per group); scale bar = 20 μm. (**E**) Quantification of Lectin positive microvessels is shown (n = 6 per group). (**F**) Immunofluorescence staining of VE-cadherin was performed to observe the changes in cardiac microvessel endothelial integrity and barrier function. scale bar = 50 μm. (**G**) Comparative quantitative analysis of fluorescence intensity of VE-cadherin (n = 6 per group). (**H**) Representative fluorescence images for intercellular adhesion molecule (ICAM)-1. scale bar = 50 μm. (**I**) Comparative quantitative analysis of fluorescence intensity of ICAM-1 (n = 6 per group). **p < 0.01

Cardiac microvascular function is closely related to myocardial function in diabetes, and the impairment of cardiac microvascular integrity and barrier function is the initial stage of vascular complications in diabetes [6, 23, 24]. Therefore, we further evaluated the effect of endothelial cell MICU1 knockout on myocardial microvascular function in 18-week-old diabetic MICU1^ecKO^ mice. Immunofluorescence staining of CD31 (green), TUNEL (red) and DAPI (blue) was performed to evaluate the apoptosis of CMECs in myocardial tissue sections of WT and MICU1^ecKO^ mice. Compared to the control mice, the myocardial tissue of diabetic mice showed more apoptosis of endothelial cells. Notably, the number of apoptotic CMECs in 18-week-old diabetic MICU1^ecKO^ mice further increased compared to age-matched diabetic mice (Fig. 3A, B, C). The microvasculature became sparse and was accompanied by a lower ratio of lectin-positive microvessels in our diabetic mouse model, which suggested decreased microvascular perfusion (Fig. 3D, E). Endothelial cell MICU1 knockout aggravated perfusion defects in diabetic MICU1^ecKO^ mice (Fig. 3D, E). Vascular endothelial (VE)-cadherin is a connexin expressed on endothelial cells and an important factor in vascular endothelial integrity and vascular permeability [25, 26]. Our data revealed that diabetes significantly downregulated VE-cadherin fluorescence in myocardial microvessels, and endothelial MICU1 knockout further inhibited the expression of VE-cadherin in diabetic MICU1^ecKO^ mice (Fig. 3F, G). Diabetes significantly upregulated ICAM-1 levels, and endothelial cell MICU1 knockout further intensified this change in diabetic MICU1^ecKO^ mice (Fig. 3H, I). Damage to the myocardial microvascular barrier and permeability promotes leukocyte migration across the endothelium, tissue edema and inflammatory reactions and leads to myocardial injury in diabetes.

### MICU1 deficiency aggravates the nitrification stress, inflammatory response and apoptosis of CMECs treated with HGHF

**Fig. 4:**
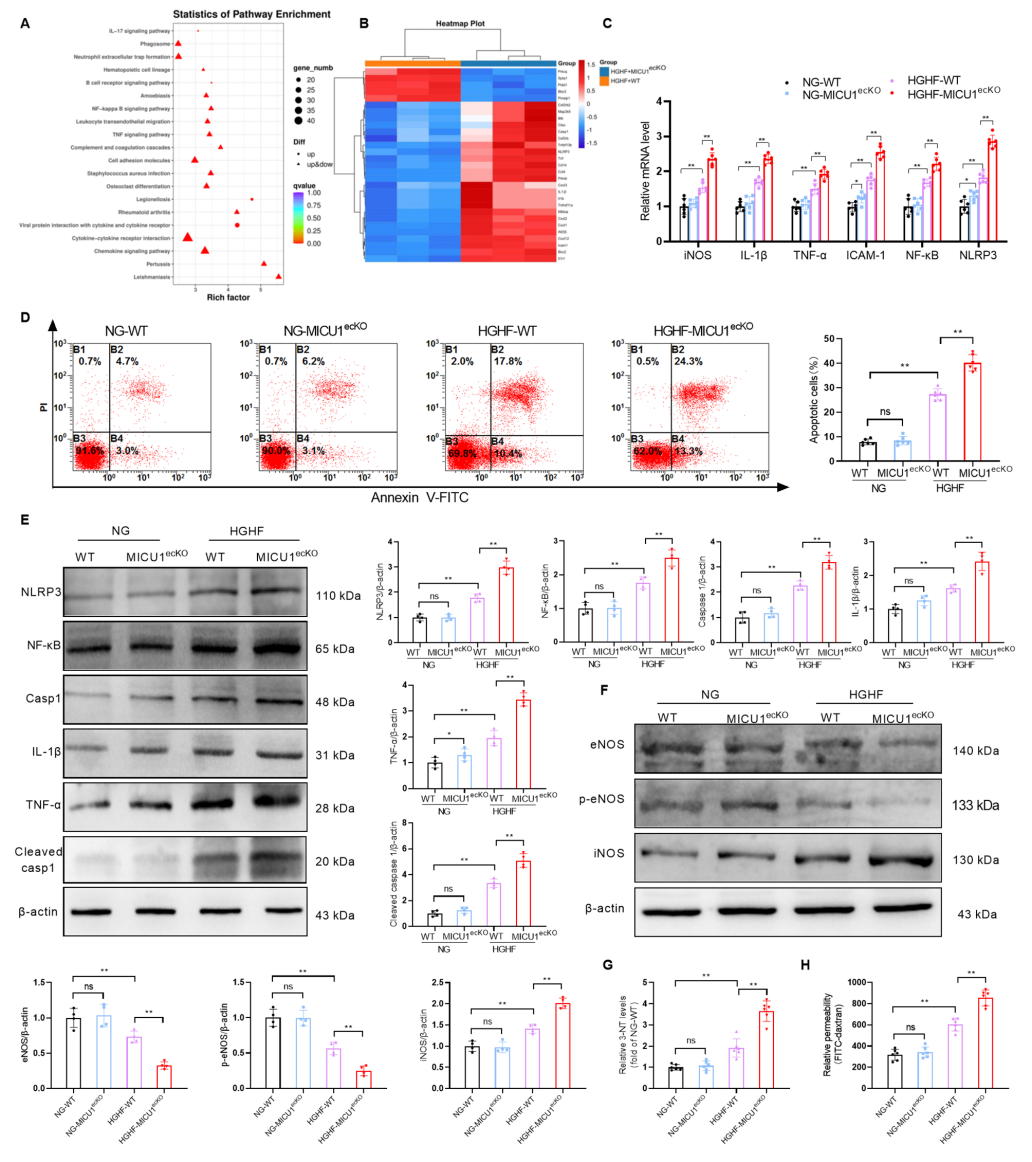
MICU1 deficiency aggravates the apoptosis, inflammatory response and nitrification stress of CMECs treated with HGHF. (**A**) KEGG pathway enrichment analysis based on these 496 overlapping DEGs. The top 10 enriched pathways ranked by the number of DEGs in the pathways are shown. (**B**) Heatmap showing the expression levels of DEGs belonging to apoptosis and the NF-κB signaling pathway in RNA-Seq. The color scale depicts the range of log2-fold changes in gene expression. (**C**) Six selected DEGs in this pathway were validated using qRT-PCR (n = 6 per group). (**D**) Flow cytometry of apoptosis using annexin V and propidium iodide (PI) staining in CMECs treated as indicated (n = 6 per group). (**E**) Changes in the expression of NLRP3, NF-κB, Caspase 1, IL-1β,TNF-α and Cleaved caspase 1as assessed by Western blotting (n = 4 per group). (**F**) Western blotting of eNOS, p-eNOS, iNOS and β-actin in CMECs (n = 4 per group). (**G**) Analysis of 3-NT Elisa results of CMECs (n = 6 per group). (**H**) FITC-dextran clearance was measured to assess changes in endothelial permeability (n = 6 per group). *p < 0.05, **p < 0.01

To further clarify the role of MICU1 in CMECs in DCM, we isolated CMECs from MICU1^ecKO^ and control mice (aged 4–5 weeks). The cells were cultured with HGHF, and transcriptomic changes were analyzed via RNA sequencing. The KEGG pathway enrichment results showed that 496 DEGs were located in 293 biological pathways, which were significantly enriched in the inflammatory response and other related pathways (Fig. 4A). Based on these results, we performed clustering analysis on the differential gene expression related to inflammatory reaction and apoptosis (Fig. 4B) and further confirmed the upregulation of iNOS, IL-1β, TNF-α, ICAM-1, NF-κB and NLRP3 by qRT-PCR in CMECs of the HGHF-MICU1^ecKO^ group (Fig. 4C). Flow cytometry showed that the apoptosis levels of CMECs treated with HGHF were significantly higher than the Con group. Endothelial cell MICU1 knockout obviously increased the apoptosis level of CMECs from the HGHF-MICU1^ecKO^ group (Fig. 4D). Western blotting showed that the expression levels of NLRP3, NF-κB, Caspase 1, IL-1β, TNF-α and Cleaved caspase 1 in CMECs of the HGHF group were significantly higher than the NG group. However, endothelial cell MICU1 knockout further exacerbated the expression levels of these inflammatory factors in CMECs of the HGHF-MICU1^ecKO^ group (Fig. 4E). We validated the RNA-sequencing results using Western blotting, and the results demonstrated that endothelial-specific knockout of MICU1 significantly decreased the protein expression of eNOS and phosphorylated eNOS (p-eNOS), increased the protein expression of iNOS in the HGHF-MICU1^ecKO^ group (Fig. 4F), which is consistent with previous qRT-PCR results (Fig. 4C). Previous studies showed that iNOS was the source of enzymes causing peroxynitrite generation [27]. The NO produced by iNOS can form peroxynitrite with superoxide. The increased expression of iNOS may lead to the reduction of NO utilization and increase the production of peroxynitrite, which causes injury of endothelial cells [28]. Because peroxynitrite frees nitrate or protein-bound tyrosine residues to generate 3-nitrotyrosine (3-NT), we observed the effect of HGHF and MICU1 knockout on endothelial cell nitrification stress using 3-Nitrotyrosine ELISA Kit for the expression of 3-NT. Studies of 3-NT showed that HGHF induced the formation of 3-NT, which was increased by endothelial cell MICU1 knockout in the HGHF-MICU1^ecKO^ group (Fig. 4G). Measurement of FITC-dextran clearance assessed the changes in endothelial permeability [29], and the data showed that endothelial cell MICU1 knockout further promoted the HGHF-induced increase in endothelial permeability (Fig. 4H). These results suggested that endothelial cell MICU1 knockout further aggravated the nitrification stress of CMECs in the myocardial microvascular endothelium in the HGHF-MICU1^ecKO^ group and promoted cell apoptosis and inflammatory reactions, which led to impairment of endothelial cell barrier function.

### MICU1 knockout exacerbates endothelial dysfunction by promoting nitrification stress mediated by mitochondrial calcium uptake in endothelial cells

**Fig. 5:**
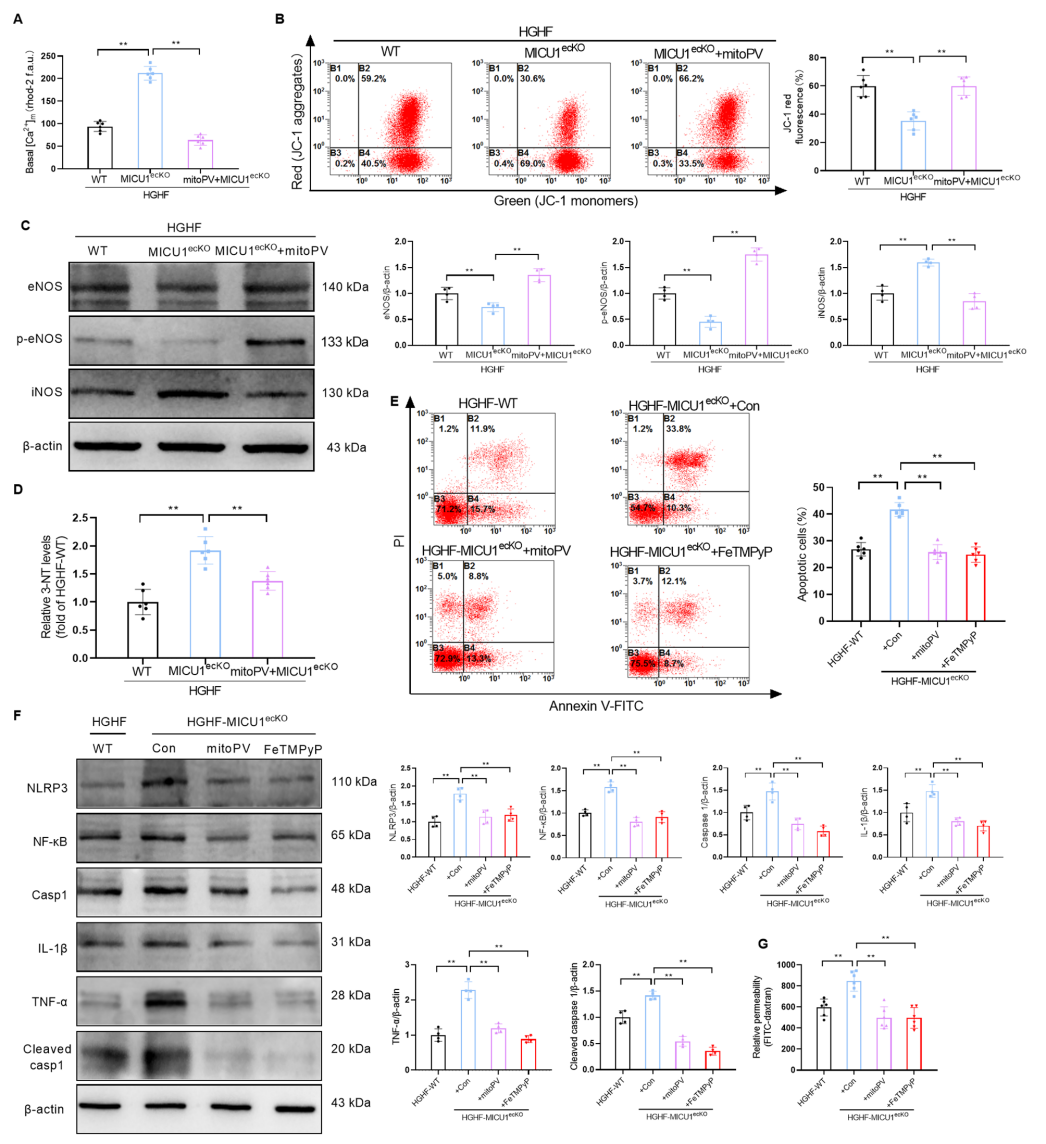
MICU1 knockout aggravates endothelial dysfunction by promoting nitrification stress mediated by mitochondrial calcium uptake in endothelial cells. (**A**) Quantitative determination of Ca^2+^ content in mitochondria of CMECs from the indicated groups (n = 6 per group). (**B**) Mitochondrial membrane potentials were analyzed using JC-1 staining in CMECs treated as indicated. Flow cytometry results are presented (n = 6 per group). (**C**). Representative Western blotting of eNOS, p-eNOS, iNOS and β-actin in CMECs (n = 4 per group). (**D**) Analysis of 3-NT Elisa results of CMECs (n = 6 per group). (**E**) Flow cytometry of apoptosis using annexin V and PI staining in CMECs treated as indicated (n = 6 per group). (**F**) Changes in the expression of NLRP3, NF-κB, Caspase 1, IL-1β, TNF-α and Cleaved caspase 1 assessed using Western blotting (n = 4 per group). (**G**) FITC-dextran clearance was measured to assess changes in endothelial permeability (n = 6 per group). **p < 0.01

Our previous studies demonstrated that upregulated MICU1 expression in diabetic myocardium promoted mitochondrial calcium uptake and reduced myocardial injury [11]. However, we measured the content of endogenous mitochondrial Ca^2+^ in isolated CMECs from MICU1^ecKO^ and control mice cultured with HGHF and found that MICU1 knockout led to a significant increase in mitochondrial Ca^2+^ in CMECs from the HGHF-MICU1^ecKO^ group (Fig. 5A). These results demonstrated that the role of MICU1 in the regulation of mitochondrial calcium homeostasis in CMECs may be significantly different from cardiomyocytes, which may be related to the basic calcium concentration in cells and mitochondria. MitoPV is a mitochondrial-targeted Ca^2+^ binding protein that can effectively removes Ca^2+^ from mitochondria. Our data indicated that endothelial cell-specific MICU1 knockout further led to the decrease in endothelial cell mitochondrial membrane potential induced by HGHF. However, mitoPV reversed the decrease in mitochondrial membrane potential caused by MICU1 knockout in CMECs compared to the HGHF-MICU1^ecKO^ group (Fig. 5B). Mitochondrial membrane potential is an important indicator of mitochondrial function. The decrease in mitochondrial membrane potential is closely related to mitochondrial nitrification stress. Based on the existing research foundation, the generation of vasoprotective nitric oxide (NO) by endothelial NO synthase (eNOS) and the contribution of inducible nitric oxide synthase (iNOS) induced by inflammatory mediators to endothelial injury and vascular dysfunction have been established. In this study, the expression levels of p-eNOS and iNOS were used to represent the protective and injurious states of endothelial cells, respectively. Based on the above-described findings, we further confirmed that mitoPV treatment reduced iNOS expression and 3-NT generation induced by MICU1 knockout, while increasing eNOS and p-eNOS in CMECs from the HGHF-MICU1^ecKO^ group (Fig. 5C, D).

We investigated the role of endothelial cell nitrification stress induced by MICU1 downregulation in diabetic myocardial microvascular injury. We tested the expression of iNOS and the production of 3-NT by regulating mitochondrial calcium. The results showed that the downregulation of MICU1 induced mitochondrial nitrification stress via mitochondrial calcium homeostasis imbalance. The flow cytometry and Western blot results further clarified that mitoPV treatment reduced endothelial cell apoptosis and inflammatory reactions induced by MICU1 knockout in the HGHF-MICU1^ecKO^ group (Fig. 5E, F). Measurement of FITC-dextran clearance assessed the changes in endothelial permeability showed that mitoPV treatment decreased the level of FITC-dextran in CMECs (Fig. 5G) and improved the endothelial barrier function of CMECs from the HGHF-MICU1^ecKO^ group. FeTMPyP is an ONOO- degradation catalyst that significantly inhibits the formation of mitochondrial nitrite. Our 3-NT Elisa experimental results confirmed this inhibition (Fig. 5D). Notably, FeTMPyP was added to CMECs to eliminate mitochondrial ONOO- production, and the inflammatory reaction, apoptosis and the level of FITC-dextran in CMECs from the HGHF-MICU1^ecKO^ group were significantly reduced (Fig. 5E, F, G). All of these findings indicated that MICU1 knockout in diabetic CMECs led to mitochondrial Ca^2+^ uptake, triggered endothelial nitrification stress, intensified inflammatory reactions and cell apoptosis, and aggravated myocardial microvascular damage.

### Upregulation of MICU1 expression alleviates HGHF-induced endothelial cell injury by inhibiting nitrification stress

**Fig. 6:**
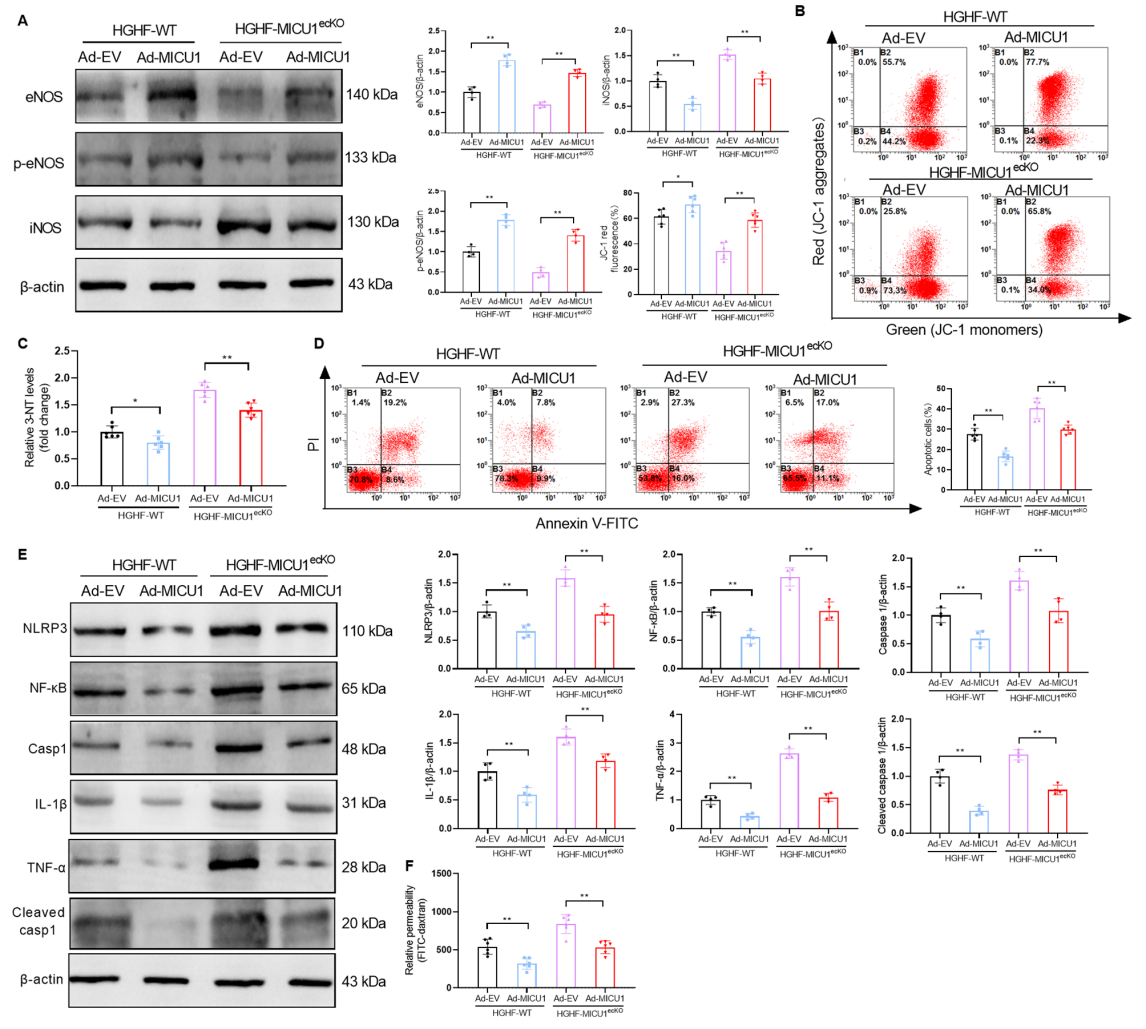
Upregulation of MICU1 expression alleviates HGHF-induced endothelial cell injury by inhibiting nitrification stress. (**A**) Representative Western blotting of eNOS, p-eNOS, iNOS and β-actin in CMECs (n = 4 per group). (**B**) Mitochondrial membrane potentials were analyzed using JC-1 staining in CMECs treated as indicated. Flow cytometry results are presented (n = 6 per group). (**C**) Analysis of 3-NT Elisa results of CMECs (n = 6 per group). (**D**) Flow cytometry of apoptosis using annexin V and PI staining in CMECs treated as indicated (n = 6 per group). (**E**) Changes in the expression of NLRP3, NF-κB, Caspase 1, IL-1β, TNF-α and Cleaved caspase 1 as assessed by Western blotting (n = 4 per group). (**F**) FITC-dextran clearance was measured to assess changes in endothelial permeability (n = 6 per group). *p < 0.05, **p < 0.01

To identify potential strategies for the treatment of diabetic cardiomyopathy, we evaluated the potential mechanisms underlying the cardioprotective effects of MICU1 in CMECs. We cultured primary CMECs with HGHF and transfected Ad-EV or Ad-MICU1 to observe the repair of endothelial cell injury. Western blotting experiments found that the forced expression of MICU1 significantly increased the protein expression of eNOS and p-eNOS, and decreased the expression of iNOS in diabetic CMECs (Fig. 6A). Based on these results, we analyzed the mitochondrial membrane potential, nitrification stress, apoptosis and inflammation of diabetic CMECs transfected with Ad-MICU1. The results showed that upregulation of MICU1 restored diabetic-diminished mitochondrial membrane potential (Fig. 6B), reduced the production of 3-NT (Fig. 6C) and apoptosis (Fig. 6D), and suppressed the inflammatory response (Fig. 6E). The results of FITC-dextran clearance also confirmed that overexpression of MICU1 attenuated HGHF-induced endothelial cell permeability injury (Fig. 6F). In summary, these results indicated that the upregulation of MICU1 reduced apoptosis and inflammatory reactions by inhibiting CMECs nitrification stress, which enhanced endothelial barrier function and reduced HGHF-induced microvascular damage.

### Cardiac endothelial-specific overexpression of MICU1 alleviates diabetes-induced cardiac microvascular injury in diabetic mice

**Fig. 7:**
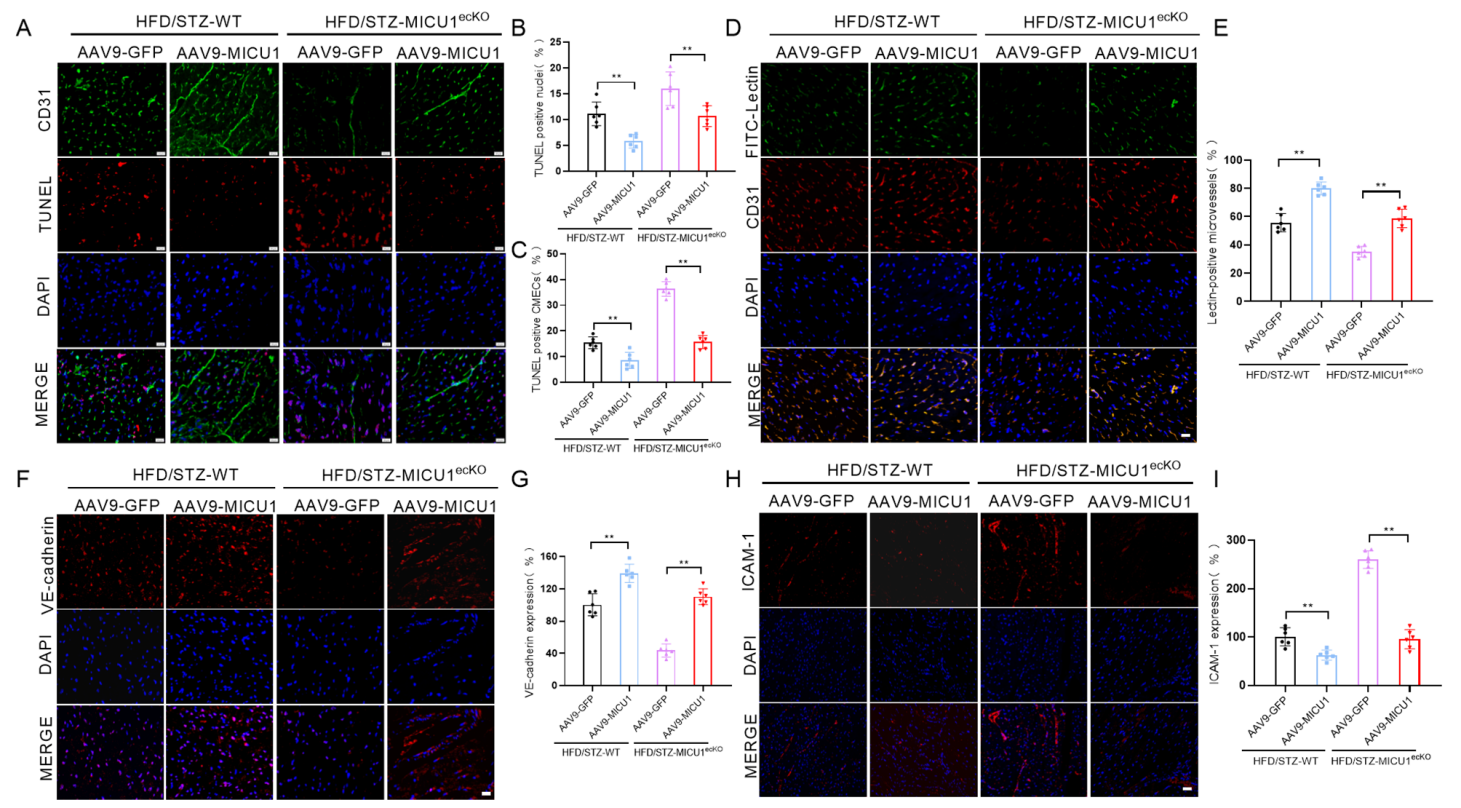
Cardiac endothelial-specific overexpression of MICU1 alleviates diabetes-induced cardiac microvascular injury in diabetic mice. (**A**) A TUNEL assay was performed to assess CMECs apoptosis, scale bar = 20 μm. (**B**) Percentages of TUNEL-positive nuclei over total number of nuclei (n = 6 per group). (**C**) Percentages of TUNEL positive CMECs over total number of CD31-positive nuclei (n = 6 per group). (**D**) Cardiac microvascular perfusion is indicated by lectin-positive microvessels (green) to CD31-positive microvessels (red), scale bar = 20 μm. (**E**) Quantification of Lectin positive microvessels is shown (n = 6 per group). (**F**) Immunofluorescence staining of VE-cadherin was performed to observe changes in cardiac microvessel endothelial integrity and barrier function, scale bar = 50 μm. (**G**) Comparative quantitative analysis of fluorescence intensity of VE-cadherin (n = 6 per group). (**H**) Representative fluorescence images of intercellular adhesion molecule ICAM-1, scale bar = 50 μm. (**I**) Comparative quantitative analysis of fluorescence intensity of ICAM-1 (n = 6 per group). **p < 0.01

We further clarified whether the upregulation of MICU1 expression in CMECs protected the cardiac microvasculature against DCM in diabetic MICU1^ecKO^ and control mice. AAV9-GFP and AAV9‐MICU1 were intramyocardially injected into 14-week-old diabetic mice (fed with a high fat diet for 6 weeks after successfully establishing the model). The expression of MICU1 was measured 4 weeks after injection, and cardiac microvascular perfusion and other functions were completed 4 weeks after injection. Western blotting (Fig. S3A, B) showed that the expression of MICU1 in CMECs was significantly increased compared to controls. Immunofluorescence staining showed that endothelial-specific overexpression of MICU1 inhibited DCM-induced apoptosis in both total cells and CMECs (Fig. 7A, B, C), improved microvascular barrier function, and ameliorated vascular permeability in myocardial tissue sections of diabetic mice (Fig. 7D-I). The same trend was observed after the expression of MICU1 was reconstructed in diabetic MICU1^ecKO^ CMECs (Fig. 7D-I). Taken together, these data indicated that the reconstruction of MICU1 expression in CMECs had a better effect on the improvement of myocardial microvascular injury in 18-week-old diabetic mice.

### Cardiac endothelial-specific overexpression of MICU1 alleviates diabetes-induced cardiac dysfunction in diabetic mice

**Fig. 8:**
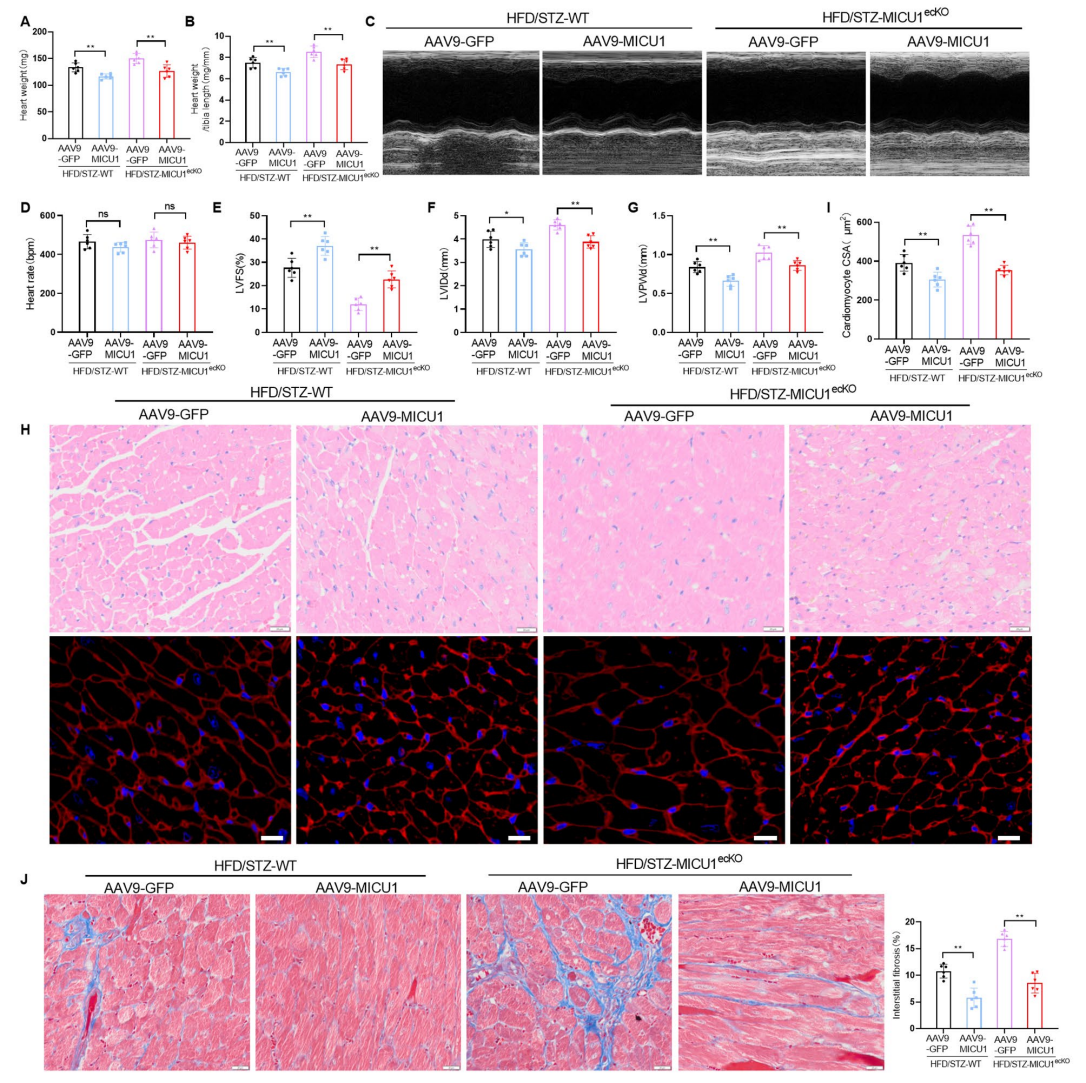
Cardiac endothelial-specific overexpression of MICU1 alleviates diabetes-induced cardiac dysfunction in diabetic mice. (**A**) The heart weight of different groups. (**B**) The ratios of heart weight to tibia length (HW/TL) for different groups. (**C**) Representative echocardiographic images of the LV in different mice (aged 18 weeks). (**D**, **E**, **F** and **G**) Echocardiographic assessment of heart rate (D), Echocardiographic assessment of LV fractional shortening (LVFS; E), LV end diastolic diameter (LVIDd; F) and LV diastolic posterior wall thickness (LVPWd; G) in different mice (n = 6, 18-week-old per group). (**H**) Cardiomyocyte cross-sectional area was analyzed using hematoxylin and eosin staining and wheat germ agglutinin staining in hearts from different mice (n = 6, 18-week-old per group); scale bar = 20 μm. (**I**) Comparative quantitative analysis of cardiomyocyte CSA. (**J**) Representative images and comparative quantitative analysis of Masson trichrome staining of hearts from different mice (n = 6 per group); scale bar = 20 μm. *p < 0.05, **p < 0.01

Heart weight and HW/TL were significantly decreased in 18-week-old diabetic MICU1^ecKO^ mice injected with AAV9-MICU1, compared to the age-matched control group injected with AAV9-GFP (Fig. 8A, B). Echocardiography showed that the cardiac function of diabetic MICU1^ecKO^ mice injected with AAV9-MICU1 was significantly improved compared to the age matched control group injected with AAV9-GFP (Fig. 8C, E, F, G), while there was no significant change in heart rate among all groups (Fig. 8D). Hematoxylin and eosin staining and wheat germ agglutinin staining showed that the cross-sectional area of cardiomyocytes in diabetic MICU1^ecKO^ mice injected with AAV9-MICU1 was significantly lower compared to the age matched control group injected with AAV9-GFP (Fig. 8H, I). Masson staining results showed that specific overexpression of MICU1 in diabetic MICU1^ecKO^ CMECs markedly reduced interstitial myocardial fibrosis (Fig. 8J). In conclusion, these data suggested that overexpression of MICU1 in CMECs significantly ameliorated myocardial fibrosis and improved left ventricular function in diabetic mice.

## Discussion

DCM is a complex disease with high morbidity and mortality. Multiple factors in DCM lead to heart failure and sudden cardiac death, but its underlying pathogenesis is not clear [30, 31]. Our previous study confirmed that the downregulation of MICU1 in diabetic cardiomyocytes mediated the disorder of mitochondrial calcium uptake and promoted DCM progression, and MICU1 upregulation, via the injection of MICU1-expressing adenovirus into the myocardium of *db/db* mice, inhibited cardiomyocyte apoptosis and improved cardiac function [11]. The role of cardiomyocyte metabolic disorder in DCM has been widely studied. However, increasing evidence shows that endothelial dysfunction may be a main mechanism in the pathogenesis of DCM [23, 24]. Long-term hyperglycemia leads to metabolic disorder and oxidative stress in CMECs, which result in a variety of effects, such as increased permeability, impaired endothelial relaxation, and reduced NO availability [32]. Therefore, it is particularly important to identify potential therapeutic targets for DCM based on CMECs.

Mitochondrial calcium homeostasis is involved in the regulation of various life processes, such as mitochondrial energy metabolism, cell autophagy, cell survival, and death [33, 34]. MICU1 in the inner membrane of mitochondria is a key regulator of mitochondrial calcium uptake and helps maintain mitochondrial calcium homeostasis [8–10]. MICU1 plays a two-way gating role in mitochondrial calcium regulation. Mallilankaraman K et al. found that MICU1 inhibited mitochondrial calcium uptake under low calcium ion conditions ([Ca^2+^] c < 3 µM) [35] but promoted calcium uptake of mitochondria under high calcium ion conditions ([Ca^2+^] c > 20 µM) [35]. Under normal physiological conditions, the concentration of cytoplasmic Ca^2+^ in CMECs is relatively low [37]. We confirmed that MICU1 knockout promoted mitochondrial calcium uptake in diabetic CMECs, which is consistent with previous studies. MICU1 plays an important role in the development of cancer, stroke, and genetic diseases, and maintaining endothelial function [38–41]. Hoffman et al. found decreased expression of endothelial MICU1 in atherosclerotic diseases, which suggests that MICU1 participates in cardiovascular injury [42]. Mallilankaraman K et al. found that silencing endothelial MICU1 promoted mitochondrial ROS production [35].

We found high expression of MICU1 in CMECs using bioinformatics database analysis, which suggested that MICU1 played an important role in myocardial microvascular function. We also confirmed that MICU1 expression in CMECs of diabetic mice decreased with the progression of DCM. Therefore, we established a diabetic mouse model using a high-fat diet in MICU1^ecKO^ mice combined with an intraperitoneal injection of STZ. We found that endothelial-specific MICU1 knockout caused disruption of myocardial microvascular barrier function in diabetic mice, exacerbated cardiac hypertrophy and fibrosis, and significantly reduced cardiac function. Notably, we found that AAV9-MICU1 specifically upregulated the expression of MICU1 in CMECs of diabetic mice, which inhibited nitrification stress, inflammatory reaction, and apoptosis of the CMECs, ameliorated myocardial hypertrophy and fibrosis, and promoted cardiac function. We isolated CMECs from MICU1^ecKO^ and control mice. Following culture in normal or HGHF conditions, we performed transcriptome sequencing and observed that the expression of nitrification stress-related iNOS enzyme and inflammation-related molecules were significantly increased in diabetic MICU1^ecKO^ CMECs. These results were verified using qRT-PCR and Western blotting experiments. These conclusions suggest that endothelial injury regulated by MICU1 is closely related to nitrification stress and inflammation.

Nitration-stressed cells generate a large amount of ONOO- within a short time, which causes protein tyrosine nitration that causes a series of damages to proteins, mitochondria, and DNA. Nitrification stress is closely related to mitochondrial function, but the specific regulatory mechanism is not clear [43, 44]. The present study detected changes in the mitochondrial membrane potential of CMECs and found that disturbances in mitochondrial calcium homeostasis mediated by MICU1 deficiency led to dysfunction of the mitochondrial respiratory chain, which increased the production of O^2−^ and a large amount of ONOO- induced by the iNOS enzyme. Elisa results showed that the production of 3-NT in CMECs of diabetic MICU1^ecKO^ mice increased significantly, which further confirmed the above conclusions. These results suggest that the imbalance of mitochondrial calcium homeostasis mediated by MICU1 knockout induces nitrification stress in CMECs. ONOO- leads to the nitration of protein lysine residues to produce 3-NT, which causes a cell damage cascade reaction and induces inflammation.

The present study found that the ONOO- scavenger FeTMPyP reduced apoptosis, inflammation, and FITC-dextran clearance in diabetic MICU1^ecKO^ CMECs, which suggest that endothelial cell injury caused by MICU1 knockout is closely related to endothelial nitrification stress mediated by mitochondrial calcium homeostasis imbalance. Our in vitro experiments found that the upregulation of MICU1 expression by Ad-MICU1 reduced the nitration products, apoptosis, and inflammation in CMECs cultured with HGHF, which indicated that MICU1 may reduce diabetes-induced endothelial cell damage by inhibiting nitrification stress. We reconstructed the expression of MICU1 in CMECs by injecting AAV9-MICU1 into the myocardium and observed a reduction in myocardial microvascular injury and an improvement in cardiac function in diabetic MICU1^ecKO^ mice. In vivo and in vitro studies confirmed the important role of endothelial MICU1 in myocardial microvascular injury in diabetes. These results improve our understanding of the pathogenesis of myocardial microvascular injury in diabetes and provide potential therapeutic targets for DCM. In conclusion, MICU1 caused nitrification stress to induce endothelial apoptosis and inflammation by disrupting mitochondrial calcium homeostasis, which led to myocardial microvascular dysfunction.

Our previous study has provided that the deficiency in mitochondrial calcium uptake due to the down-regulation of MICU1 expression in diabetes cardiomyocytes leads to myocardial oxidative damage, thereby promoting the occurrence and progression of DCM. Further investigation has revealed that restoring MICU1 expression in diabetic myocardium through myocardial injection of MICU1 adenovirus not only ameliorates cardiomyocyte apoptosis but also significantly reduces apoptosis in CMECs. Compensatory hypertrophy of cardiomyocytes and interstitial fibrosis are common pathological manifestations of non-infarct myocardial injury. The present study demonstrates that the restoration of MICU1 expression effectively mitigates CMECs injury, enhances myocardial microcirculation perfusion, and diminishes compensatory hypertrophy of cardiomyocytes and interstitial fibrosis. Consequently, in contrast to primary myocardial injury, we posit that microcirculation disorders resulting from CMEC injury exert a more substantial influence on the occurrence and progression of DCM, thereby representing a critical intervention target for DCM.

The current study has several limitations. First, we did not use tamoxifen-inducible MICU1^ecKO^ mice in our study. Before the establishment of a diabetic mouse model, the cardiac function of MICU1^ecKO^ mice was slightly decreased compared with WT mice. We will use conditional MICU1 endothelial cell-specific knockout mice to more accurately clarify the role of MICU1 in DCM in the future. Second, in this study, a diabetic model induced by a high-fat diet combined with STZ can be considered as a mixed model of type 1/type 2 diabetes. Initially, the high-fat diet induces metabolic disorder resulting in insulin resistance. Subsequently, STZ selectively impairs pancreatic islet beta cells, leading to inadequate insulin secretion. The combination of these two factors ultimately leads to hyperglycemia, which can induce organ damage. While this model does not fully replicate clinical diabetes, it effectively demonstrates the association between myocardial microvascular dysfunction and DCM in this study. Numerous previous studies have employed and acknowledged the utilization of this diabetic model. Third, this study demonstrated that the restoration of MICU1 expression in diabetic mice through AAV9-MICU1 intervention inhibited the advancement of DCM. However, it remains inconclusive whether this therapeutic effect is solely attributed to the direct influence of MICU1 or if it is a secondary consequence of improved diabetes. Additionally, our investigation revealed that MICU1 overexpression enhanced the glucose tolerance of diabetic mice (Figure S2), indicating that MICU1 overexpression directly contributes to the amelioration of DCM, while also benefiting from the concurrent improvement of diabetes. Further research is required to elucidate the underlying mechanism by which MICU1 improves diabetes.

## Conclusions

In summary, we found that MICU1 expression was downregulated in the CMECs of diabetic mice. Overexpression of endothelial MICU1 reduced nitrification stress-induced apoptosis and inflammation by inhibiting mitochondrial calcium uptake, which improved myocardial microvascular function and inhibited DCM progression. MICU1 regulates endothelial nitrification stress by maintaining mitochondrial calcium homeostasis, which is one mechanism in this process. In conclusion, our results suggest that myocardial endothelial MICU1 is a therapeutic target for DCM.

### Electronic supplementary material

Below is the link to the electronic supplementary material.

Supplementary Material 1

## Data Availability

All data generated or analyzed during this study are included in this published article.
