# Supplementary material for: Endothelial MICU1 alleviates diabetic cardiomyopathy by attenuating nitrative stress-mediated cardiac microvascular injury
Source: Cardiovasc Diabetol. 2023 Aug 17;22:216. doi: 10.1186/s12933-023-01941-1 (PMC10436431; doi:10.1186/s12933-023-01941-1)
Supplement: Supplementary file 1 — Supplementary Material 1 [file 12933_2023_1941_MOESM1_ESM.docx]

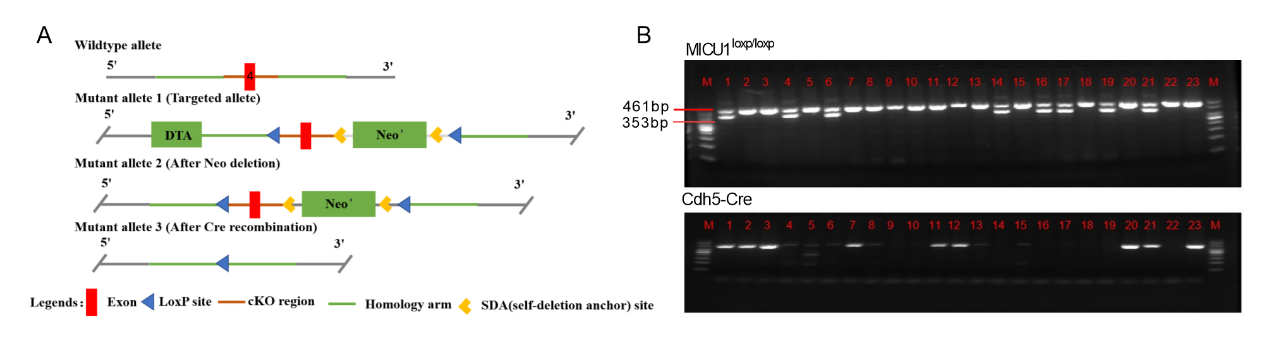


**Figure S1** Schematic representation of the construction strategy for MICU1^loxp/loxp^ mice. **A** Genotyping Strategy of MICU1^loxp/loxp^ mice. **B** The PCR products from Cdh5-cre and MICU1^loxp/loxp^ mice tail samples were identified by agarose gel electrophoresis.WT: 353 bp, MT: 461bp. DNA marker: 50 bp DNA ladder.


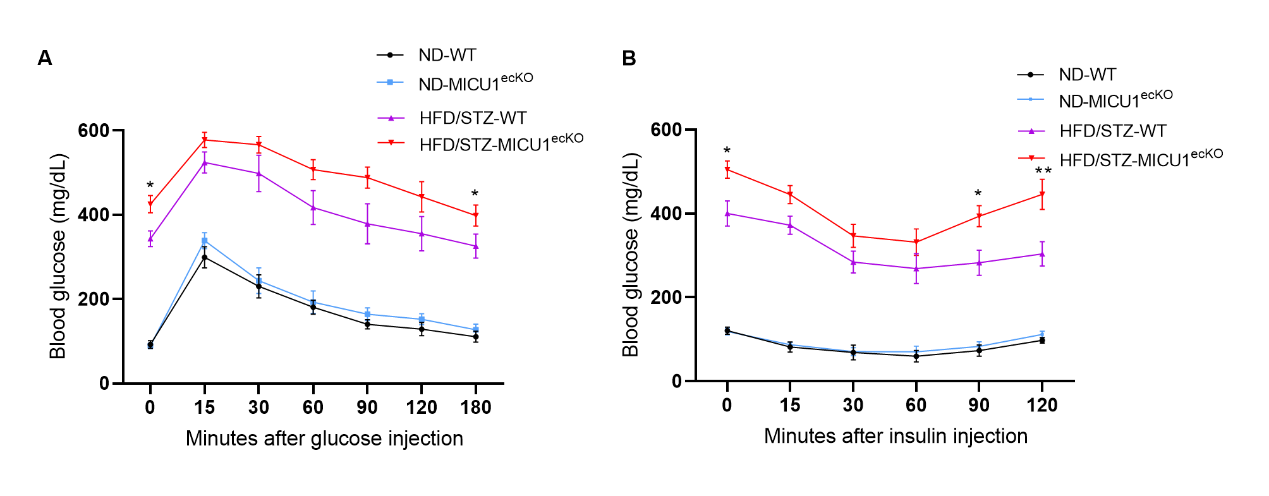


**Fig. S2** Intraperitoneal glucose tolerance tests (IPGTTs) and insulin tolerance tests (ITTs) were used to detect plasma blood glucose levels in mice. **A** Raw data of intraperitoneal glucose tolerance tests (IPGTTs; 2g/kg glucose) in different groups of mice (n = 6 per group). **B** Raw data of intraperitoneal insulin tolerance tests (ITTs；0.75 units/kg insulin) in different groups of mice (n = 6 per group). **p* < 0.05, ***p* < 0.01.


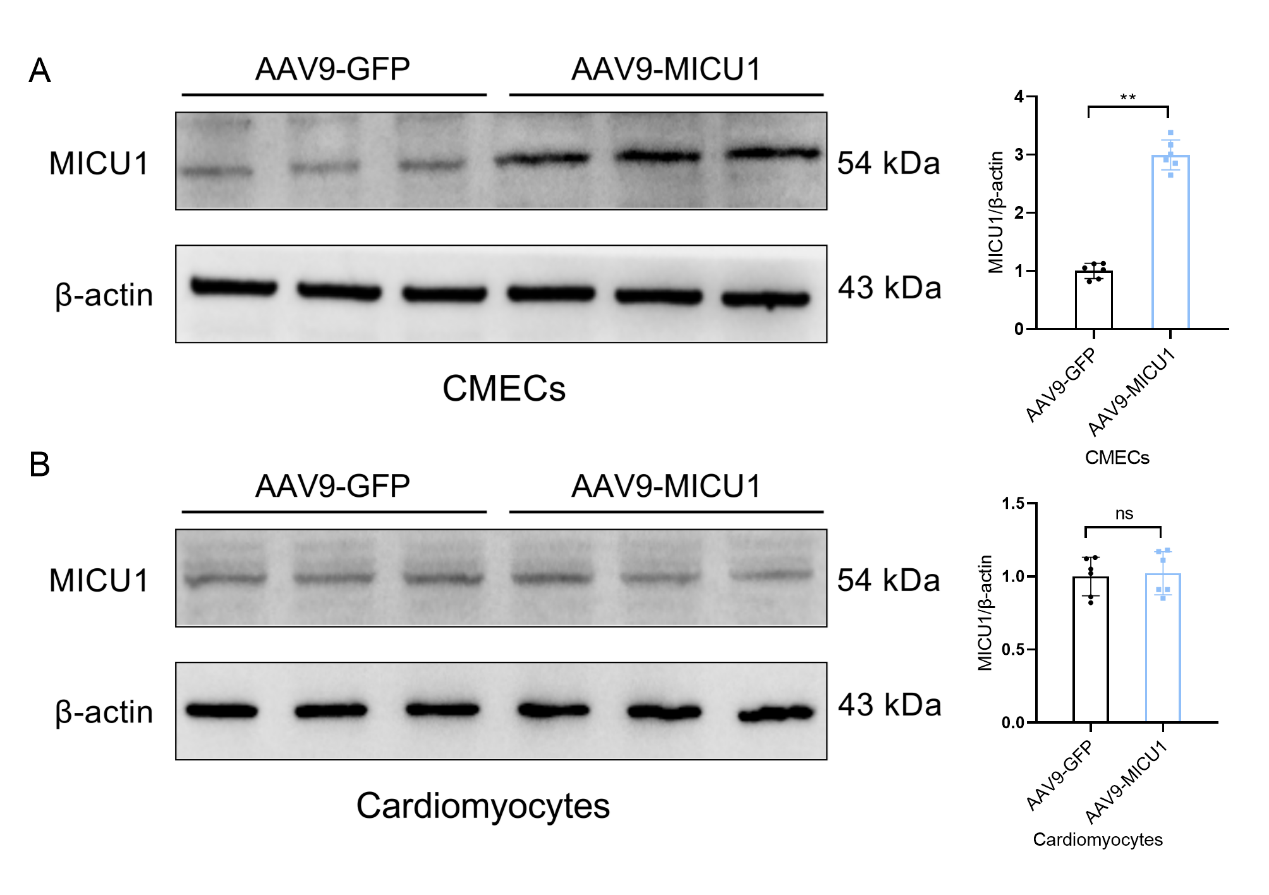


**Fig. S3** AAV9 transfection efficiency in the cardiac microcirculation was detected by western blotting. **A** Western blot analysis was used to quantify MICU1 expression in CMECs isolated from AAV9-GFP or AAV9-MICU1 infected heart. **B** Western blot analysis was used to quantify MICU1 expression in cardiomyocytes isolated from AAV9-GFP or AAV9-MICU1 infected heart (n = 6 per group). ***p* < 0.01.
